# Evaluation of DNA methylation biomarkers *ASCL1* and *LHX8* on HPV-positive self-collected samples from primary HPV-based screening

**DOI:** 10.1038/s41416-023-02277-z

**Published:** 2023-04-26

**Authors:** Lisanne Verhoef, Maaike C. G. Bleeker, Nicole Polman, Renske D. M. Steenbergen, Renée M. F. Ebisch, Willem J. G. Melchers, Ruud L. M. Bekkers, Anco C. Molijn, Wim G. Quint, Folkert van Kemenade, Chris J. L. M. Meijer, Johannes Berkhof, Daniëlle A. M. Heideman

**Affiliations:** 1 Amsterdam UMC, Location Vrije Universiteit Amsterdam, Pathology, De Boelelaan 1117, Amsterdam, The Netherlands; 2 Cancer Center Amsterdam, Imaging and Biomarkers, Amsterdam, The Netherlands; 3 Radboud University Medical Center, Obstetrics and Gynecology, 6525 GA Nijmegen, the Netherlands; 4 Radboud University Medical Center, Medical Microbiology, 6525 GA Nijmegen, the Netherlands; 5 GROW School for Oncology and Developmental Biology, Maastricht University, 6229 ER Maastricht, the Netherlands; 6 Catharina Hospital, 5623 EJ Eindhoven, the Netherlands; 7 Eurofins NMDL-LCPL, 2280 CA Rijswijk, the Netherlands; 8 Erasmus MC University Medical Center, Pathology, 3015 GD Rotterdam, the Netherlands; 9 Amsterdam UMC, Location Vrije Universiteit Amsterdam, Epidemiology and Data Science, De Boelelaan 1117, Amsterdam, The Netherlands

**Keywords:** Cervical cancer, Cancer epigenetics

## Abstract

**Background:**

Host-cell DNA methylation analysis can be used to triage women with high-risk human papillomavirus (HPV)-positive self-collected cervicovaginal samples, but current data are restricted to under-/never-screened women and referral populations. This study evaluated triage performance in women who were offered primary HPV self-sampling for cervical cancer screening.

**Methods:**

Self-collected samples from 593 HPV-positive women who participated in a primary HPV self-sampling trial (IMPROVE study; NTR5078), were tested for the DNA methylation markers *ASCL1* and *LHX8* using quantitative multiplex methylation-specific PCR (qMSP). The diagnostic performance for CIN3 and cervical cancer (CIN3 + ) was evaluated and compared with that of paired HPV-positive clinician-collected cervical samples.

**Results:**

Significantly higher methylation levels were found in HPV-positive self-collected samples of women with CIN3 + than control women with no evidence of disease (*P* values <0.0001). The marker panel *ASCL1/LHX8* yielded a sensitivity for CIN3 + detection of 73.3% (63/86; 95% CI 63.9–82.6%), with a corresponding specificity of 61.1% (310/507; 95% CI 56.9–65.4%). The relative sensitivity for detecting CIN3+ was 0.95 (95% CI 0.82–1.10) for self-collection versus clinician-collection, and the relative specificity was 0.82 (95% CI 0.75–0.90).

**Conclusions:**

The *ASCL1/LHX8* methylation marker panel constitutes a feasible direct triage method for the detection of CIN3 + in HPV-positive women participating in routine screening by self-sampling.

## Background

Primary screening for high-risk (hr) human papillomavirus (HPV) provides better protection against cervical intraepithelial neoplasia grade 3 (CIN3) and cervical cancer (CIN3+) than cervical cytology [1, 2]. Consequently, many cervical cancer screening programmes nowadays include primary HPV testing. The Netherlands converted to HPV testing with cytology triage in 2017. HPV testing as a primary screening tool offers the opportunity to explore self-sampling as an alternative to clinician sampling for all women invited for screening. Self-sampling is a promising strategy to overcome barriers to cervical cancer screening and to increase coverage [3]. Furthermore, self-sampling has gained increased interest during the COVID-19 pandemic [4]. HPV self-sampling has demonstrated similar clinical accuracy as HPV testing on clinician-collected samples [5, 6]. However, cytology triage currently requires recalling women who are HPV-positive on a self-collected sample for clinician-based sampling. Emerging evidence has demonstrated that the detection of host-cell DNA methylation represents a promising alternative triage strategy [7, 8], with the potential of being directly applicable to self-collected screening samples.

Aberrant DNA methylation is an epigenetic hallmark of cancer [9]. DNA hypermethylation of tumour suppressor genes is an early and frequent molecular alteration in cervical carcinogenesis [10]. DNA methylation levels of various host-cell genes have been reported to increase with CIN grade and are highest in cervical cancer [8]. Recent studies have shown that virtually all cervical cancers are methylation positive [11, 12]. Within the group of CIN2/3 lesions, it was found that lesions associated with a long-lasting (≥5 years) hrHPV infection have significantly higher methylation levels compared with lesions with a more recently acquired (<5 years) hrHPV infection [13, 14]. Based on these findings, it is assumed that methylation positivity of specific host-cell genes is characteristic of CIN lesions with a high short-term risk of cancer, referred to as advanced CIN2/3 lesions [15]. In addition, the absence of methylation was found to be associated with the regression of CIN2/3 lesions [16, 17]. In view of the above, DNA methylation biomarkers have emerged as a promising triage tool for HPV-based cervical cancer screening to specifically detect advanced CIN lesions in need of treatment. A meta-analysis on the triage performance of various methylation markers in HPV-positive clinician-collected cervical samples reported a pooled sensitivity for CIN3 + of 71.1% (95% CI 65.7–76.0) at a predefined specificity of 70% [8].

There is a growing number of studies reporting on DNA methylation analysis for the direct triage on HPV-positive self-collected samples [18–23]. However, variable diagnostic performances have been reported, which highlights the need for further investigation. To date most studies have been performed in cohorts of underscreened or never-screened women and referral populations. The current study aimed to assess the triage performance of host-cell methylation analysis on HPV-positive self-collected samples within the context of routine HPV-based screening. For this, we used self-collected samples of HPV-positive women participating in the IMPROVE study, a primary HPV self-sampling trial carried out within the organised population-based screening programme in the Netherlands [5]. The IMPROVE study cohort enabled us to compare methylation data between self-collected samples and paired clinician-collected cervical samples and cervical tissue specimens from the same HPV-positive women. We evaluated the methylation markers *ASCL1* and *LHX8* that were discovered in self-collected samples [19] and previously evaluated for clinical performance on clinician-collected cervical samples [24, 25] and self-collected samples from screening non-attendees [19].

## Methods

### Clinical specimens

This is a post hoc analysis of the IMPROVE study (Netherlands Trial Register, number NTR5078), a randomised non-inferiority trial, that was performed to evaluate the clinical accuracy of HPV testing on self-collected samples and clinician-collected samples within the setting of the Dutch cervical cancer screening programme. A detailed description of the IMPROVE trial has been previously published [5]. In brief, 16,410 women were enrolled and randomised (1:1) to the intervention group (self-sampling) or the control group (clinician-based sampling). Consistent with the randomised, paired screen-positive design, HPV-positive women (*n* = 1020) were retested using the other collection method. In accordance with the current guidelines of the Dutch primary HPV screening programme, women with positive HPV test results were triaged by cytology. HPV-positive women with baseline borderline or mild dyskaryosis (BMD) or worse (≥BMD) as per CISOE-A classification equal to ASC-US or worse (≥ASC-US) in the Bethesda classification [26], were immediately referred to a gynaecologist for colposcopy. HPV-positive women with normal cytology (i.e., negative for intraepithelial lesion or malignancy [NILM]) at baseline were advised to undergo repeat cytological testing after 6 months and were referred for colposcopy when repeat cytology was ≥BMD. Women with two consecutive normal cytology results (also referred to as 2x NILM) were referred to the next routine screening round at a 5-year interval.

**Fig. 1:**
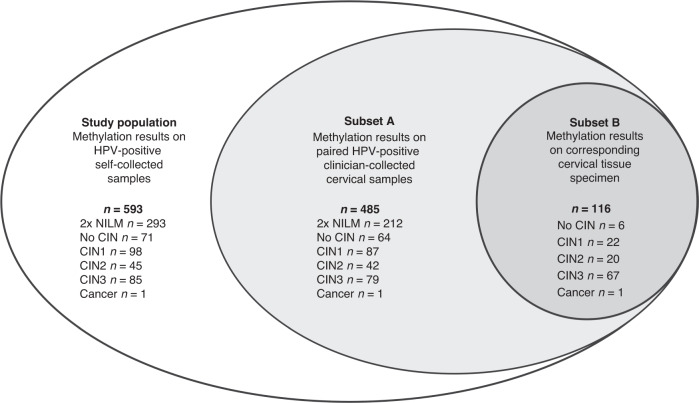
Venn diagram showing the relation between the sample series used in this study. Data analysis comprised methylation results of self-collected samples from 593 HPV-positive women (white ellipse; study population), methylation results of paired clinician-collected samples from 485 (out of 593) women (light-grey ellipse; subset A) and methylation results of corresponding formalin-fixed paraffin-embedded (FFPE) tissue samples of 116 (out of 485) women (dark grey ellipse; subset B). HPV human papillomavirus, *n* number of, NILM negative for intraepithelial lesion of malignancy, CIN cervical intraepithelial neoplasia.

For this study, we used self-collected samples from HPV-positive women, who either had a histology endpoint or two consecutive normal cytology results, and who provided consent for follow-up research (*n* = 780). Of these, 187 samples were excluded due to insufficient leftover material for valid methylation analysis, resulting in a final study population of 593 HPV-positive self-collected samples with *ASCL1/LHX8* methylation results (Fig. 1). *ASCL1/LHX8* methylation data on paired HPV-positive clinician-collected cervical samples were available for 485 women (Fig. 1, subset A) [25]. These women were HPV-positive on both their clinician-collected and their self-collected sample [5]. In a subgroup of 116 women (Fig. 1, subset B), the corresponding formalin-fixed paraffin-embedded (FFPE) tissue sample was additionally available for methylation analysis. This study was approved by the Medical Ethics Committee of Amsterdam UMC, Vrije Universiteit Amsterdam (Amsterdam, The Netherlands; METC 2018/09, TcB 2018.106). The IMPROVE study was approved by the Ministry of Public Health (The Hague, The Netherlands; IMPROVE VWS no. 2014/32).

### DNA isolation

DNA from self-collected samples was isolated using the NucleoMag 96 Tissue kit (Macherey-Nagel, Düren, Germany) and a Microlab Star robotic system (Hamilton, Gräfelfing, Germany), according to the recommendations of the manufacturer. Whole tissue sections from the FFPE tissue blocks were prepared using the sandwich method. The first and last sections were stained with haematoxylin and eosin (H&E) to check for the presence of lesions. In-between sections were collected in sterile PCR tubes for DNA isolation. DNA was isolated using the QIAamp DNA FFPE tissue kit (Qiagen, Hilden, Germany) according to the manufacturer’s instructions and eluted with easyMAG 3 elution buffer (bioMérieux, Boxtel, The Netherlands). DNA concentrations were measured using a Qubit fluorometer (Qubit, ThermoFisher Scientific, Waltham, MA, USA).

### Host-cell DNA methylation analysis

*ASCL1/LHX8* methylation analysis was performed, blinded for cytology and histology outcomes, by quantitative methylation-specific PCR (qMSP) on bisulphite-converted DNA, essentially as described by Snellenberg et al. [27]. DNA was subjected to sodium bisulphite treatment using the EZ DNA Methylation Kit (D5002, Zymo Research, Irvine, CA, USA), according to the manufacturer’s instructions. Bisulphite-converted DNA was subsequently used as input for qMSP analysis of the *ASCL1* and *LHX8* genes. In the multiplex qMSP, the housekeeping gene β-actin (*ACTB*) was used as a reference to ensure successful bisulphite conversion and sample quality. The methylation levels of *ASCL1* and *LHX8* were normalised to *ACTB* using the quantification cycle (Cq) value (2^−ΔΔCq^ × 100) to obtain ΔΔCq ratios [28].

### Data and statistical analysis

The original cytology and histology results were retrieved from pathology laboratories through the Dutch Nationwide Pathology Databank (PALGA) [29]. For analysis, the CISOE-A classification was translated into the Bethesda nomenclature [26]. Histology was categorised as no CIN, LSIL/CIN1, HSIL/CIN2, HSIL/CIN3 (further referred to as CIN1, CIN2 and CIN3), or invasive cervical cancer, according to the latest WHO classifications [30]. Adenocarcinoma in situ (AIS) and carcinoma in situ (CIS) were included in the group of CIN3 lesions. Data on HPV were retrieved from the study database [5], with HPV genotype information available for HPV16, -18, -31, -33, -35, -39, -45, -51, -52, -56, -58, -59, -66 and -68.

All statistical analyses and visualisations were performed using the square root-transformed ΔΔCq ratios of *ASCL1* and *LHX8*. Methylation levels of each marker were categorised in sextiles and visualised in relative frequency histograms per disease category (2x NILM, no CIN, CIN1, CIN2, CIN3 and cervical cancer). The Kruskal–Wallis omnibus test was applied to calculate differences in continuous DNA methylation levels among disease categories, with post hoc testing using the Mann–Whitney *U* test. Spearman’s rank correlation coefficient was used to analyse correlations between methylation levels in paired self-collected samples, clinician-collected cervical samples and cervical tissue specimens. Differences in methylation levels between paired sample types were assessed using the Mann–Whitney *U* test.

The sensitivity, specificity, positive predictive value (PPV), and negative predictive value (NPV) for the detection of CIN3 + were determined with Wald 95% confidence intervals (95%CI) for the following triage strategies: methylation analysis, HPV16/18 genotyping, HPV16/18 genotyping combined with methylation analysis and cytology. For comparison, relative CIN3 + sensitivity and specificity were determined together with 95% CIs. The methylation status of *ASCL1* and *LHX8* was labelled positive when Cq was below 40. The *ASCL1/LHX8* marker panel was considered positive if at least one of the markers tested positive (“believe-the-positive”) [31]. Genotyping results were available for 491 women, and HPV16/18 genotyping as triage strategy was labelled positive if HPV16 and/or HPV18 were present. HPV16/18 genotyping combined with methylation analysis was labelled positive if HPV16 and/or HPV18 were present or the *ASCL1/LHX8* marker panel was positive. Cytology results of the paired clinician-collected cervical sample were available for 593 women and cytology as triage was labelled positive if baseline cytology on the paired clinician-collected cervical sample was ≥ASC-US (i.e., ≥BMD). The association between methylation and age was studied using a logistic regression analysis. Methylation data on paired self-collected and clinician-collected cervical samples were compared, by calculating overall agreement and Cohen’s kappa and by calculating relative CIN3 + sensitivity and relative specificity together with 95% CIs.

Statistical analyses were performed using SPSS software for Windows (version 26.0, SPSS Inc., Chicago, IL, USA) and GraphPad Prism (V9.1.0).

## Results

### Methylation of *ASLC1* and *LHX8* in HPV-positive self-collected samples

**Fig. 2:**
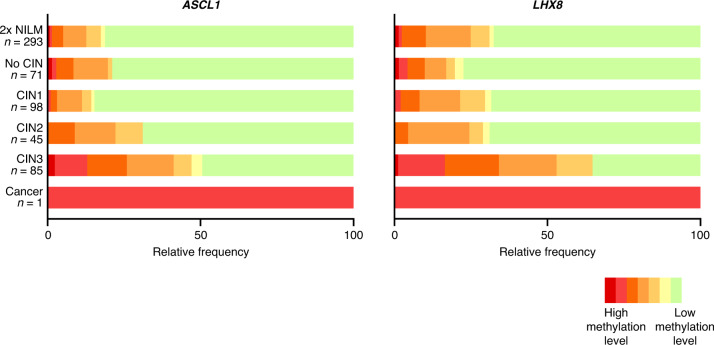
Stacked histograms showing the relative frequency of methylation levels of *ASCL1* and *LHX8* per disease category. Methylation levels were categorised in sextiles. CIN cervical intraepithelial neoplasia, 2x NILM two consecutive normal cytology results.

A total of 593 self-collected samples from HPV-positive women who participated in the IMPROVE study (median age 40.0; IQR 34–49; range 29–60) were included for analysis of the DNA methylation markers *ASCL1* and *LHX8* (Fig. 1). The series comprised of one woman with cervical squamous cell carcinoma, 85 women with CIN3, 45 women with CIN2, and 462 control women who had no evidence of CIN2+, including 293 women with two consecutive normal cytology results, 71 with no CIN and 98 with CIN1. The methylation levels of each marker for each disease category are shown in Fig. 2. The methylation levels of *ASCL1* and *LHX8* in HPV-positive self-collected samples increased with the severity of underlying cervical disease (Kruskal–Wallis omnibus test, both *P* values <0.0001). A significant increase in methylation levels was observed for both *ASCL1* and *LHX8* in self-collected samples from women with CIN3+ compared to self-collected samples from control women (Mann–Whitney *U* test, both *P* values <0.0001).

**Fig. 3:**
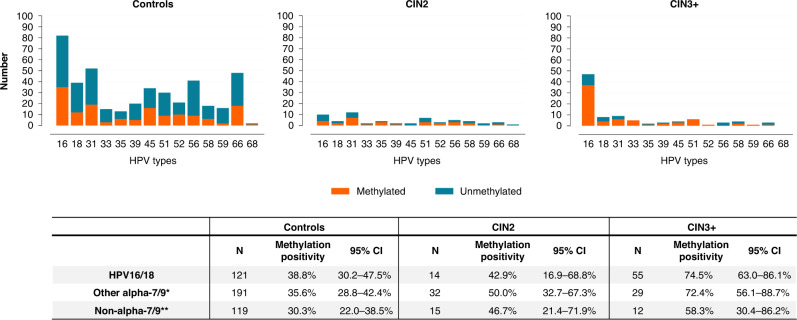
*ASCL1/LHX8* marker panel outcome according to HPV genotype stratified for disease category. Multiple HPV infections were counted as separate attributions. HPV human papillomavirus, CIN cervical intraepithelial neoplasia, N total number of samples, CI confidence interval. *Other alpha-7/9 types: HPV31, 33, 35, 39, 45, 52, 58, 59 and 68. **Non-alpha-7/9 types: HPV51, 56 and 66.

The ability of the individual markers and their combination to distinguish CIN3 + in HPV-positive self-collected samples is reported in Table 1. The *ASCL1/LHX8* marker panel demonstrated a CIN3 + sensitivity of 73.3% (95% CI 63.9–82.6%) with a corresponding specificity of 61.1% (95% CI 56.9–65.4%). The CIN3 + sensitivity and specificity did not change with age for both the individual markers and the marker panel (*P* value = 0.162 and 0.377 for *ASCL1* and *LHX8*, respectively, and *P* value = 0.147 for the marker panel *ASCL1/LHX8). ASCL1/LHX8* marker panel outcome in relation to HPV genotype is shown in Fig. 3. Table 1 also reports the performance characteristics of HPV16/18 genotyping, HPV16/18 genotyping with *ASCL1/LHX8* methylation analysis, and cytology triage. The relative sensitivity of the *ASCL1/LHX8* marker panel versus HPV16/18 genotyping for CIN3 + was 0.98 (95% CI 0.82–1.17) and the relative specificity 0.92 (95% CI 0.83–1.01). The combination of HPV16/18 genotyping with *ASCL1/LHX8* methylation analysis showed a CIN3 + sensitivity of 88.9% (95% CI 81.6–96.1%) with a corresponding specificity of 57.0% (95% CI 52.3–61.8%). The relative sensitivity of the combination of HPV16/18 genotyping with *ASCL1/LHX8* methylation analysis versus cytology triage on a paired clinician-collected cervical sample for CIN3 + was 1.00 (95% CI 0.89–1.13) and the relative specificity 0.83 (95% CI 0.75–0.92).

**Table 1:** Clinical performance for the detection of CIN3+.

	Specificity			Sensitivity			PPV		NPV	
	*n/N*	%	95% CI	*n/N*	%	95% CI	%	95% CI	%	95% CI
*ASCL1* methylation	408/507	80.5%	77.0–83.9%	44/86	51.2%	40.6–61.7%	30.8%	23.2–38.3%	90.7%	88.0–93.4%
*LHX8* methylation	351/507	69.2%	65.2–73.2%	56/86	65.1%	55.0–75.2%	26.4%	20.5–32.3%	92.1%	89.4–94.8%
Marker panel *ASCL1/LHX8*	310/507	61.1%	56.9–65.4%	63/86	73.3%	63.9–82.6%	24.2%	19.0–29.4%	93.1%	90.4–95.8%
HPV16/18 genotyping	286/419	68.3%	63.8–72.7%	53/72	73.6%	63.4–83.8%	28.5%	22.0–35.0%	93.8%	91.1–96.5%
HPV16/18 genotyping with marker panel *ASCL1/LHX8*	239/419	57.0%	52.3–61.8%	64/72	88.9%	81.6–96.1%	26.2%	20.7–31.7%	96.8%	94.6–99.0%
Cytology*	346/507	68.2%	64.2–72.3%	78/86	90.7%	84.6–96.8%	32.6%	26.7–38.6%	97.7%	96.2–99.3%

*CI* confidence interval, *n* number of, *N* group size, *NPV* negative predictive value, *PPV* positive predictive value.

*On paired clinician-collected cervical sample (threshold ASC-US).

### Methylation levels in paired self-collected samples, clinician-collected samples and tissue specimens

Paired *ASCL1/LXH8* methylation data on self-collected and clinician-collected samples were available for 485 HPV-positive women (Fig. 1, subset A), including one woman with cervical squamous cell carcinoma, 79 women with CIN3, 42 women with CIN2, and 363 controls. The overall agreement in *ASCL1/LXH8* methylation outcomes between self-collected and clinician-collected samples was 64.1% (310/507), 95% CI 59.7–68.4% (Cohen’s kappa 0.250). The CIN3 + sensitivity and specificity of *ASCL1/LHX8* methylation analysis were lower in self-collected samples compared to clinician-collected cervical samples, with a relative sensitivity of 0.95 (95% CI 0.82–1.10) and a relative specificity of 0.82 (95% CI 0.75–0.90).

**Fig. 4:**
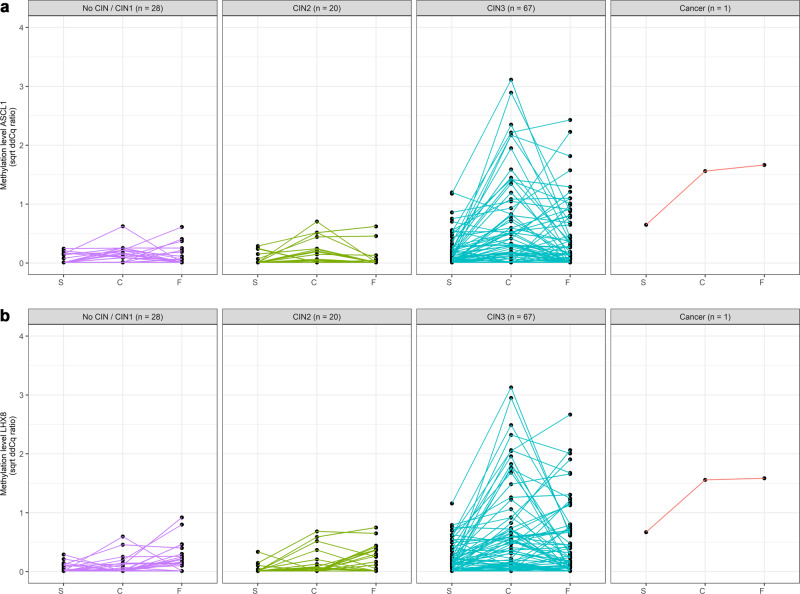
Conditional scatterplots displaying the methylation levels for *ASCL1* and *LHX8* for paired self-collected samples (S), clinician-collected cervical samples (C) and cervical tissue specimens (F) stratified for histology. Differences in methylation levels of (**A**) *ASCL1* and (**B**) *LHX8* between paired sample types were assessed using the Mann–Whitney *U* test: self-collected samples compared to clinician-collected cervical samples, both *P* values <0.0001; self-collected samples compared to cervical tissue specimens, *P* value = 0.134 and 0.002, respectively; and clinician-collected cervical samples compared cervical tissue specimens, *P* value = 0.130 and 0.179, respectively. Cq quantification cycle, CIN cervical intraepithelial neoplasia, sqrt square root.

For a small subset of women (Fig. 1, subset B, *n* = 116), paired cervical tissue specimens were also available for methylation analysis. The series comprised of one woman with squamous cell carcinoma, 67 women with CIN3, 20 women with CIN2, 22 women with CIN1 and 6 women with no CIN. Paired methylation data stratified for histology are visualised in Fig. 4. A moderate correlation between the methylation levels of both *ASCL1* and *LHX8* in different sample types was observed (Spearman’s Rho 0.563 and 0.550 for self-collected samples versus clinician-collected samples, 0.459 and 0.359 for self-collected samples versus tissue specimens and 0.524 and 0.429 for clinician-collected samples versus tissue specimens, respectively). The methylation levels of *ASCL1* and *LHX8* in self-collected samples were lower compared to those in paired clinician-collected cervical samples (both *P* values <0.0001).

## Discussion

In this study, we showed that methylation levels of *ASCL1* and *LHX8* in HPV-positive self-collected samples correlated with underlying disease severity and were significantly higher in women with CIN3 + than in control women with no evidence of disease. The CIN3 + sensitivity of the *ASCL1*/*LHX8* marker panel on HPV-positive self-collected samples was 73.3% (95% CI 63.9–82.6%) at a specificity of 61.1% (95% CI 56.9–65.4%). The triage performance of *ASCL1/LHX8* methylation analysis on self-collected samples was somewhat lower than that on clinician-collected cervical samples (relative sensitivity for CIN3 + detection 0.95, 95% CI 0.82–1.10 and relative specificity 0.82, 95% CI 0.75–0.90). Our data indicate that the methylation marker panel *ASCL1/LHX8* constitutes a feasible direct triage method for the detection of CIN3 + in HPV-positive women participating in routine screening by self-sampling. The advantage of DNA methylation analysis as a triage test is the use of an objective, non-morphological assay, with a high reproducibility [32], directly applicable to self-collected samples. Importantly, about half of the women with ≥ASC-US on the paired clinician-collected cervical sample taken for cytology triage, and ~75% of the women with histological samples of CIN3+ could have been directly referred for colposcopy, without recall for clinician-collection, after *ASCL1/LHX8* methylation analysis on the HPV-positive self-collected sample.

In our study, the performance of the bi-marker panel *ASCL1/LHX8* did not differ from that of HPV16/18 genotyping. Of interest, methylation positivity rate did not differ across the various genotypes, supporting the continued value of host-cell DNA methylation markers in the post-vaccination era [33]. HPV16/18 genotyping and *ASCL1/LHX8* methylation analysis were to a certain extent complementary in line with previous findings [16, 34]. Although comparison with cytology on the paired clinician-collected sample must be done with caution due to the fact that the HPV-positive women in the IMPROVE study were managed based on cytology, HPV16/18 genotyping in combination with the bi-marker panel *ASCL1/LHX8* increased CIN3+ sensitivity to a level that did not differ from cytology triage on a clinician-collected cervical sample, though at the cost of a decreased specificity. This combined strategy nonetheless obviates the need for a recall for a clinician-collected cervical sample for cytology triage, which may counterbalance the increased referral rate due to the drop in specificity, and integrates high adherence to triage. The total impact of screening on the detection of cervical lesions is not only related to the performance of the test, but also dependent on compliance with follow-up procedures. In the Netherlands, about 10–20% of women with an HPV-positive self-collected sample do not show up for cytology triage [35]. Therefore, alternative triage methods, such as host-cell DNA methylation analysis either or not combined with HPV genotyping, may be considered which do not require an extra visit to the clinician. The decrease in the specificity of the combined strategy is expected to gradually fade when HPV-vaccinated women will enter the screening programme. The first vaccinees will reach screening age in the Netherlands in 2023. It should furthermore be noted that the performance of cytology as triage strategy is largely dependent on the quality of cytology, which varies widely among countries and is high in the Netherlands. The full molecular strategy directly applicable to self-samples would be particularly beneficial in settings without a quality-assured cytology infrastructure or low-resource settings where cytology screening is limited.

The unique study design of the IMPROVE study allowed for a direct comparison of methylation analysis on self-collected and clinician-collected samples from the same HPV-positive women. Though promising, the results also highlight that the performance for implementation of the methylation assay in an organised screening setting as a single triage test on self-collected samples would require further improvement. Alike for cytology triage which currently requires a retest at 6–12 months in case of normal cytology at baseline to ensure sufficient protection, one could consider to improve the NPV by offering repeat testing to those women that screen HPV-positive but methylation negative at baseline testing of their self-collected sample. Alternatively, depending on country preferences, a strategy with baseline testing only could comprise recall of women that screen HPV-positive but methylation negative on their self-collected sample for an additional cervical sample at the clinician’s office for e.g., methylation analysis or cytology testing. For the current study cohort, these latter algorithms would have resulted in NPVs of 96.1% (95% CI 93.5–98.8%) and 98.6% (95% CI 97.1–100.2%), respectively (data not shown).

The methylation markers used in this study were discovered using a genome-wide screen on self-collected samples of non-attendees [19]. In an initial validation series, these markers showed good clinical performance for CIN3 detection in both HPV-positive lavage (sensitivity 74%; specificity 79%) and brush (sensitivity 88%; specificity 81%) self-collected samples from screening non-attendees [21]. The *ASCL1*/*LHX8* marker panel also demonstrated good triage performance on HPV-positive clinician-collected cervical samples [24, 25]. Notably, in all studies, samples from women with cervical cancer were positive for the methylation marker panel. Recently, also the utility of methylation testing of *ASCL1* and *LHX8* for the detection of CIN3+ in urine has been demonstrated [36].

To the best of our knowledge, this is the first study evaluating methylation markers in self-collected samples from women who were offered primary HPV self-sampling for cervical cancer screening. The relatively low sensitivity and specificity for CIN3+ of *ASCL1/LHX8* in primary HPV self-sampling may be related to the study population. Previous studies have mainly been performed on self-collected samples in underscreened and never-screened women and referral populations [19–23, 34], which may not fully represent the women who attend the population-based screening. In these populations, the incidence of CIN3 is higher than in a population of screening attendees [37]. Moreover, due to screening at regular intervals, CIN3 lesions identified by population-based screening have a relatively short duration since onset, are relatively small, and are known to have lower methylation levels [14, 16, 22]. Furthermore, cervicovaginal self-collected samples have a different cellular composition and proportion of cervical cells compared to clinician-collected cervical samples. Technical advances in DNA methylation analysis to allow more sensitive and specific assessment in low-input samples will be of interest to optimise the methylation-based triage of self-collected samples [38]. A low-input may be circumvented in routine setting by use of a larger fraction of the self-sample in the methylation assay. This was not possible in our study given the setting of using restricted leftover sample material. Furthermore, direct cell conversion protocols and automated solutions for methylation analysis offer the ability to improve performance and provide a solution for the high-throughput application of methylation assays in cervical cancer screening [39, 40].

The strengths of this study include the large sample size and the use of samples from a primary self-sampling trial conducted within the setting of the Dutch cervical cancer screening. Although the study protocol slightly differed from the current national screening protocol for primary HPV self-sampling, our study demonstrates direct triage utility of host-cell DNA methylation markers with satisfactory performance on HPV-positive self-collected screening samples. These data are particularly relevant for settings where cytology is not routinely available. Another strength is the IMPROVE study design, which allowed the comparison of methylation data on HPV-positive self-collected samples to paired clinician-collected cervical samples and cervical tissue specimens. Our findings on the agreement are in line with previous data comparing methylation marker performance in paired samples, reporting moderate to the good agreement [16, 18, 20, 22, 23, 41, 42]. A limitation of our study is that our results could have been affected by verification bias. We did not have a histology endpoint for HPV-positive women with two consecutive normal cytology results, given that these women were referred to the next screening round in accordance with the current guidelines of the Dutch screening programme. Nonetheless, this effect seems minimal as HPV**-**positive women with two consecutive normal cytology results have a very low risk of CIN3+.

In conclusion, the *ASCL1/LHX8* methylation marker panel, alone or in combination with genotyping, constitutes a feasible direct triage method for detecting CIN3+ in HPV-positive women participating in routine screening by self-sampling. Our results support further clinical validation in prospective screening studies using HPV self-sampling.

## Data Availability

The data that support the findings of our study are in the anonymised form available from the corresponding author upon reasonable request and following the data protection regulations.
